# Women’s health and psychological well-being in the return-to-work process after long-term sick leave for common mental disorders: women’s and first-line managers’ perspectives

**DOI:** 10.1186/s12889-024-20350-x

**Published:** 2024-10-15

**Authors:** Åsa Hedlund, Marja-Leena Kristofferzon, Eva Boman, Karen Nieuwenhuijsen, Annika Nilsson

**Affiliations:** 1 Department of Caring Sciences, University of Gävle, Kungsbäcksvägen 47, Gävle, 801 76 Sweden; 2 Department of Occupational Health Science and Psychology, University of Gävle, Gävle, Sweden; 3 Amsterdam UMC, Department of Public and Occupational Health, location University of Amsterdam, Amsterdam Public Health research institute, Meibergdreef 9, Amsterdam, The Netherlands

**Keywords:** Common mental disorders, First-line managers, Health, Psychological well-being, Return to work, Women

## Abstract

**Background and aim:**

Common mental disorders are common reasons for long-term sick leave, especially among women. Return to work is often complex and unsuccessful, why more knowledge is needed regarding women’s health and psychological well-being in the return-to-work process. Therefore, the aim was to describe women’s health and psychological well-being in the return-to-work process, from women’s and first-line managers’ perspectives.

**Methods:**

Individual interviews were conducted with 17 women and 16 first-line managers. Qualitative content analysis was performed based on the content areas “Women’s health (i.e. overall well-being, both physical and psychological) throughout the whole RTW process” and “Women’s psychological well-being (happiness, meaning and a sense of being significant) at work after work resumption” Themes and categories were created.

**Results:**

Women and managers had similar descriptions, i.e. that women’s health and psychological well-being depend on the individual characteristics of women themselves, their private life, work and other stakeholders. However, women described relational work tasks (e.g. meeting patients) as beneficial for health, and highlighted small stressors in the work environment, which the managers did not. Having work that was compatible with private life, being in good health, having stimulating work tasks and strengthening relationships at work were important for the women’s psychological well-being.

**Conclusions:**

Based on women’s and first-line managers experiences, promotion of women’s health and psychological well-being during the return-to-work process requires individually adapted assessments and actions involving women’s entire life situation. First-line managers should know that relational work tasks (e.g., meeting patients) can be beneficial for women’s health as well as that minor stressor in the work environment can put their health at risk.

## Background

Common mental disorders (CMDs), i.e., stress-related disorders, depression and anxiety, affect a large part of the working population worldwide [1–3]. In this population, women are most affected [4]. CMDs lead to longer sick leave periods than physical diagnoses do [5]. The return-to-work (RTW) process after CMDs is often complex and is associated with an increased risk of relapse [6, 7]. It is known that improved health is important for RTW (e.g. work resumption) [8] and that psychological well-being at work may be important for staying at work in the long run [9]. Despite this, research has mainly focused on RTW per se or the effects of psychological therapies on symptoms. By investigating experiences of health and psychological well-being in women with CMDs in the RTW process, from both women’s and first-line managers´ perspectives, RTW stakeholders and RTW researchers may gain a more comprehensive and nuanced understanding of women’s way back to work after long-term sick leave for CMDs.

**Fig. 1:**
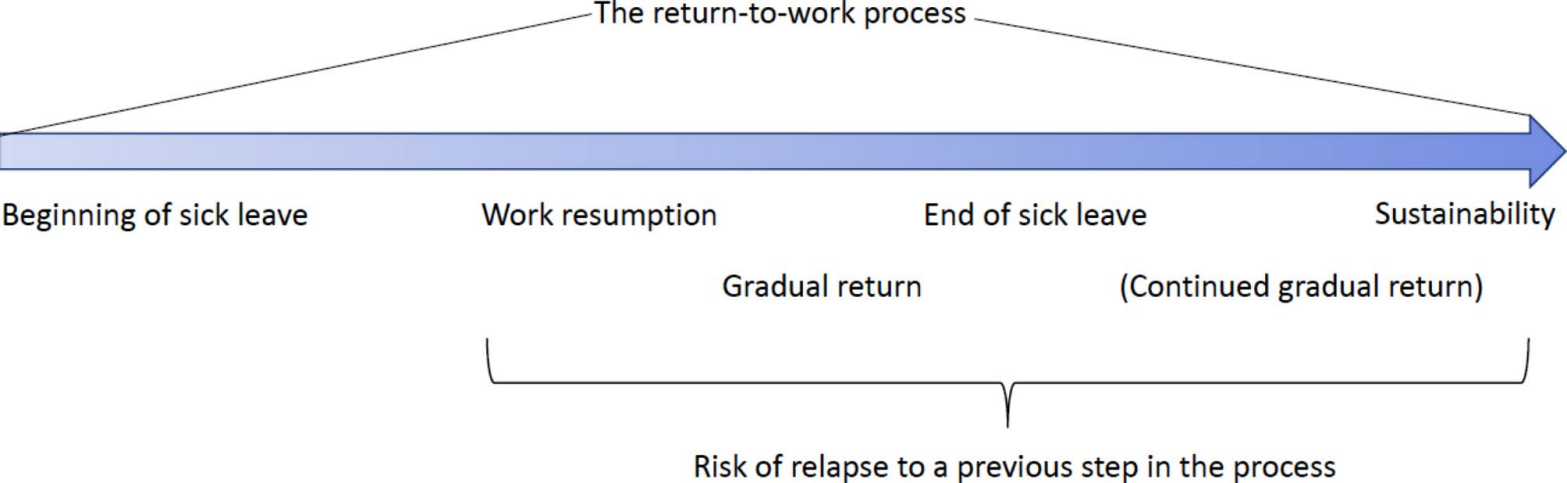
The administrative phases of the return-to-work process

CMDsare the most common causes of long-term sick leaves (> 60 days) among women in Sweden [10]. There is a need for gender specific research in the area of CMD and RTW, because women and men often face different conditions in work- and private life [11, 12]. In Sweden, as in many other European countries, sectors such as healthcare and education have a high level of gender segregation with a clear majority of female workers [10–13]. Female dominated professions tend to have more emotional stressors and organizational deficits than other occupations [11]. In addition, during the RTW-process women experience more demands on their personal lives than men, such as taking care of family members and organizing social gatherings [12]. The RTW process is often complex because the employee’s and employer’s needs must be aligned over time, despite changes in the employee’s performance profile [14]. A common definition of the RTW process is that it extends from the first day of sick leave through work resumption, a gradual increase of working degree and eventually to sustainability at work [15–17], see Fig. 1. Sustainability can be defined as the employee working for some time (e.g., a couple of weeks or months) after the sick leave has ended [18, 19] and as having developed strategies to manage daily life [15]. However, he/she might still be at risk of relapse [20]. In Sweden, sick leave is based on a medical statement from a treating physician. The statement is assessed by the Social Insurance Agency, which determines whether the individual is eligible for sickness benefits. The return usually takes place with an increase of 25% in contract hours at a time [21]. The RTW process does not always follow a linear pattern, because, within the administrative stages of the RTW process, employees are struggling with their process of health improvement [22, 23].

Health is usually considered to be a subjective feature that includes how an individual generally feels and functions in daily life [24, 25]. In human sciences, it is often seen to also include general well-being [26–28]. Furthermore, symptoms of a disease or disorder are related to the individual’s health [27, 29]. Women in the RTW process after CMDs have reported that decreased symptom burden is important to their ability to begin and continue working [30]. The importance of decreased CMD symptoms for making progress in the RTW process was also found in a meta-analysis by Fisker et al. [8]. Psychological treatments are often used for reducing symptom burden and facilitating RTW among individuals on sick leave for CMDs, but according to systematic reviews, the effect of these is small and not particularly sustainable [11, 31]. Therefore, more knowledge about symptoms in relation to the RTW process is needed, for example, how symptoms are expressed over time [8]. As described above, health involves more than symptoms, one example being perceived general health. A study of women on long-term sick leave for CMDs reported low perceived general health at baseline [30], but in a 1-year follow-up, this failed to predict RTW (i.e., increased working degree) [29]. However, in Nielsen et al. study [32], a better perceived general health was able to predict a shorter time to end of sickness benefits among individuals with CMDs. Hence, it seems that health plays a significant role in the RTW process, but we need a better understanding of how it appears and is managed over time. Most research about general health has been quantitative. There is a need for more qualitative research regarding general health because that reveals the content of it and therefore enables stakeholders to support the individuals adequately [33].

After work resumption, positive feelings about work might be important for staying at work in the long run. Employees with musculoskeletal disorders described how positive feelings at work, such as joy or feeling that work is energizing, made them stay at work despite the presence of symptoms [34]. In line with this, Fasbender et al. [35] found that high job satisfaction was associated with lower turnover rates among nurses. Therefore, positive feelings at work may facilitate a sustained RTW. This is beneficial for the employee and for society, as it allows valuable expertise to be retained in the labour market. Positive feelings about work can be referred to as psychological well-being at work – a phenomenon that is receiving increased attention in RTW research [9]. Psychological well-being refers to the individual’s emotions or feelings [36, 37], such as happiness [36, 38], meaning [36, 37, 39] and the sense of being significant in one’s context [38–40]. However, to date, there is a lack of evidence concerning what contributes to psychological well-being at work after long-term sick leave for CMDs. Hedlund et al. [30] did find that among women going through the RTW process after CMDs, experiences of meaning at work and belonging to the social context at work are important advantages while working [30]. The first-line manager may have a key role in this.

First-line managers play an important role in promoting health and psychological well-being at work for their employees [41–44]. In the RTW process, this can be done by adjusting the work situation, consulting occupational health stakeholders, and making employees feel included in the social context at work [42]. In Sweden, first-line managers are obligated to create a good organizational and psychosocial work environment characterized by more than only the absence of risks and ill health. It should for example also include opportunities for personal development, variation and social contacts [44]. Supporting employees in the RTW process after CMDs is often complicated according to first-line managers. Scharf et al. [14] found that managers experience having insufficient knowledge of CMDs and, therefore, do not know how to adequately support returning employees. International and Swedish studies revealed that managers feel a lack of knowledge about CMDs and a lack of support in the RTW process, causing for example insecurity and arbitrary management of employee’s needs [14, 45, 46, 47]. Moreover, almost half of the managers focus on a rapid RTW rather than health, after they perceive that the employees’ first critical phase is over [46]. A focus on solely RTW can be experienced as a pressure rather than a support according to women on long-term sick leave for CMDs [30]. Furthermore, discrepancies between managers and employees in their perceptions of the RTW-support provided to the employee have been found to prolong the RTW process [48]. It is therefore, important to identify these discrepancies. Even though there are some studies among first-line managers in relation to RTW after CMDs, there is a lack of studies focusing on employees’ health and psychological well-being in the RTW process from first-line managers’ perspectives.

To sum up, previous research has shown that health and psychological well-being are important aspects to investigate deeper in the RTW process after CMDs. By including the perspectives of both the returning women and first-line managers, a more comprehensive picture can be drawn and potential discrepancies be identified. The present study aimed to describe experiences of women’s health and psychological well-being in the RTW process, from women’s and first-line managers’ perspectives.

## Methods

### Recruitment and participants

Women were selected for this qualitative descriptive study using convenience sampling via Facebook groups for different occupations (nurses, teachers etc.) and the discussion forum Familjeliv.se. Administrators for the Facebook groups/forum were asked for permission to post a request for interest. Those interested in participating contacted the first author and received the information letter and the text for consent via e-mail. Then they returned the text for consent and agreed on a time and place (face-to-face, online or by telephone) for the interview. To be included in the study, women had to have been on sick leave for 2–12 months for a stress-related condition, depression, and/or anxiety syndrome, had ended their sick leave during the past 1–9 months and were working at least 50% at the time of the interview. Of the 45 interested women, 28 were excluded because they, for example, were still on part-time sick leave or had relapsed before the interview.

The first-line managers were selected from two counties in central Sweden via purposive sampling based on the occupations in which the women worked, i.e., health care, social care and so on. Operations managers (“second-line” managers) were contacted in the first instance with a request for interest in the study. Geographical variation and inclusion of both public and private sector operations were sought. The inclusion criteria were having worked as a first-line manager for at least six months and having experienced being the supervisor of women going through the RTW process after CMDs. The operational managers could choose to forward the invitation to their first-line managers or ask the researchers to contact them directly. The first-line managers that were interested in participating contacted the first author via e-mail and received an information letter and text for written consent: 16 managers were interested and all of them fulfilled the inclusion criteria. The managers and the researcher (ÅH) agreed on a time and place for the interview.

In total, the study included 33 participants: 17 women and 16 first-line managers from urban or rural areas. The women were between 29 and 53 years old (mean 42.4). They had been on sick leave between 2.5 and 23 months (mean 9.2) and ended their sick leave 1–8 months ago (mean 3.5). All women had been on full-time sick leave from the beginning. The most commonly reported cause of sick leave was work-related issues, often in combination with private life issues. The managers were between 35 and 63 years old (mean 48.1) and had worked between 9 months and 17 years as first-line managers (mean 6.4 years), at the same or different places. Additional participant characteristics are presented in Tables 1 and 2.

**Table 1:** Characteristics of the women (self-reported)

Variables	*n* (17)
Sector^a^	
Healthcare^b^	13
Social care	3
Schools	1
Diagnoses^c^	
Stress-related disorders	10
Combination of several common mental disorders	4
Depression	3
Percentage of full-time work at the time of the interview	
100%	8
50–95%	9
Number of children at home^d^	
Zero	5
One	4
Two	6
Three or more	2
Marital status	
Married/ Living with a partner	14
Single	3
Returned to former job or another	
Former job	10
Another^e^	7

a. The vast majority worked within the public sector

b. Two had administrative jobs within healthcare

c. Bad working conditions alone or in combination with a heavy burden in private life was the most commonly mentioned cause of the CMD/sick leave

d. Part-time or full-time, biological or partner’s child

e. In two cases involuntary because the former workplace was shut down or the employment ended. All women except for one worked in the same kind of job as before they changed jobs

**Table 2:** Characteristics of the first-line managers (self-reported)

Variables	*n* (16)
Sector^a^	
Healthcare	11
Social care	4
Schools	1
Gender	
Woman	14
Man	2
Leadership Education	
Yes	13
No	3
Number of employees	
<20	1
20–29	3
30–39	6
40–49	5
>50	1

a. The vast majority worked within the public sector. Two first-line managers had the same operational manager

### Data collection

Two semi-structured interview guides were created, one for the women and one for the first-line managers, including two areas: “Women’s health throughout the whole RTW process” and “Women’s psychological well-being at work after work resumption”. Health concerns well-being in general, i.e., both physical and psychological. Psychological well-being at work concerned a feeling of happiness, meaning and a sense of being significant at work. The interview questions are presented in Tables 3 and 4. All interviews also included background questions such as age and profession, and all were audiotaped.

**Table 3:** Women’s interview guide

Questions	
Questions concerning health during the whole RTW-process: 1. Describe how you have experienced your health and well-being during sick leave and after having returned to work?	
2. What symptoms were most difficult to deal with during sick leave and after having returned to work?	
3. Has your health changed from the time you went on sick leave until today? If so, in what way?	
4. Describe your health today.	
5. What is needed for your health to improve/be maintained?	
6. How do you think your manager notices that your health has improved/worsened?	
7. What parts of your job are easy?	
8. Are there any obstacles in your work today that make it difficult/impossible for you to perform your work tasks? If so, describe what is difficult or impossible at work.	
9. During this period, what have you learned about how you feel? Questions about psychological well-being after work resumption:	
10. What is needed for you to feel joy in your work? *	
11. What is needed for you to feel like a significant person at work? *	
12. What is needed for you to feel meaning in your work? * Final questions:	
13. Do you have anything else to add that you think is important? 14. Based on your experience of having returned to work after long-term sick leave for mental ill health, what would you like us to ask managers?	

*Psychological well-being at work after work resumption

**Table 4:** First-line managers’ interview guide

Questions	
Questions concerning women’s health during the whole RTW-process: 1. Describe your experiences, as a manager, of promoting the health and well-being of women during and after their long-term sick leave for mental ill health.	
2. Which of the women’s health problems have you found most challenging for you, as a manager, to deal with during their sick leave/return to work?	
3. What do you think women on long-term sick leave for mental ill health need for their health to improve/be maintained?	
4. Some women find it easier to return to work after long-term sick leave for mental ill health than others do. What do you think this depends on?	
5. What health-related challenges remain for women who have returned to work?	
6. What are your thoughts about the returning woman’s needs compared with those of someone who has been on sick leave for physical problems? *	
7. What are the conditions you feel you need in order to promote the health and well-being of those returning to work after sick leave for common mental disorders? ^*^	
8. What additional knowledge do you feel you need in order to promote the health and well-being of those returning to work after sick leave for common mental disorders? * Questions about women’s psychological well-being after work resumption:	
9. Do you have any experience of what is important women to feel: - joy in their work? - meaning in their work? - significant as a person in their work? Describe these experiences. Final question:	
10. Do you have anything else to add that you think is important?	

*Questions suggested by the women

To cover the RTW process for the women, some questions were retrospectively formulated while others dealt with the present. Two pilot interviews were conducted: One before the data collection and one at the beginning of the data collection. Only the latter was included in the study, as the former was carried out prior to ethical approval with a person known to ÅH. No changes were made to the interview guide based on the pilot interviews. The interviews were carried out between March and September 2022 using digital conference tools (*n* = 15) or the telephone (*n* = 2), and they lasted between 31 and 69 min (mean 50). After the interviews, the interviewed women were asked to suggest additional questions for the managers’ interview guide. The authors discussed the questions and grouped those that were similar, resulting in two groups: “Experience/knowledge” and “Conditions”. This resulted in three additional questions being added to the managers’ interview guide (see Table 4).

The first-line managers’ interview guide covered the same two areas as the women’s interview guide. Interviews were carried out between September and October 2022 using video conference tools and lasted between 23 and 40 min (mean 29). The first two interviews were pilot interviews, and both were included in the study.

### Data analyses

The women’s and first-line managers’ data were analysed separately using inductive content analysis [49]. The two areas in the interview guides – “Women’s health throughout the whole RTW process” and “Women’s psychological well-being at work after work resumption” – were treated as content areas in the analyses and were therefore analysed separately. The women’s data were analysed before the managers’ data. All interviews were transcribed verbatim. The questions to the first-line managers, from the women, were included in the data analysis together with the other questions. Subsequently, meaning units related to the study aim were identified, condensed, and abstracted into codes that made sense in relation to the study questions. The codes were then sorted based on similarities and differences. Because data regarding the content area “Women’s health throughout the whole RTW process” answered the question “How?”, subthemes emerged by looking for threads of meaning in the condensed meaning units. Data regarding the content area “Women’s psychological well-being at work after work resumption” answered the question “What?”, and therefore categories were created based on the sorted codes [49]. When women’s and first-line managers’ data had been analysed separately, it appeared that similar subthemes and categories had been identified in both groups. Hence, the subthemes and categories were merged and slightly reformulated to cover both the women’s and the managers’ experiences. Regarding the first content area, one theme emerged based on the shared essence of meaning in the subthemes. To enhance credibility, the research group discussed subthemes, themes, and categories several times throughout the analysis process. The analyses were carried out in Microsoft Word by sorting the material into digital tables.

## Results

The results of the analysis of the women’s and the first-line managers ' data are based on the two content areas: “Women’s health throughout the whole RTW process” and “Women’s psychological well-being at work after work resumption”. Results from the first content area are represented by one theme and four subthemes, and results from the second content area are represented by three categories; see Table 5. Quotations are inserted in the text and marked with quotation marks and italics.

**Table 5:** Overview of content areas with the underlying theme, subthemes and categories for the women and the first-line managers

Content area	Women’s health during the RTW process
Theme	Struggling with limited resources in an unpredictable situation and approaching stability
Sub-themes	Experiencing difficulties in syncing health improvement with the degree of work
	Trying to find a match between work characteristics and the person
	Having to depend on supporters’ personality and experience
	Relying on women’s use of internal resources

Content area	Women’s psychological well-being at work after work resumption
Categories	Stable personal conditions
	Stimulating work tasks
	Strengthening relationships at work

### Women’s health during the RTW process: Struggling with limited resources in an unpredictable situation and approaching stability

This content area concerned the women’s experiences of their health, and the first-line managers’ experiences of promoting women’s health, during the period from the beginning of sick leave to after the sick leave had ended. The theme describes both how the women struggled to improve their own health and how the managers struggled to improve the women’s health, as well as how both groups experienced limited resources to do this. They also felt it was an unpredictable situation because every person involved is unique, and adding the environment to this creates a unique situation. Over time, the women’s health improved, but in most cases not to “good” levels.

#### Experiencing difficulties in syncing health improvement with the degree of work

The women and managers described that the process of women’s health improvement went up and down and that it felt fragile, lengthy, problematic to control and for the women, also emotional. At the beginning of the sick leave period, the women experienced severe symptoms such as exhaustion, cognitive difficulties, and panic attacks. Because of this, they felt being on sick leave was a necessity, even though it involved feelings of shame and guilt in relation to work and family. However, sick leave did not always provide the best conditions for healing, for example when sick leave periods were short or when they waited for the Insurance Agency’s decision on sickness benefits: *“…then the doctor told me*,* ‘well*,* I did everything I could so that you… I wrote everything I could so that you could stay on sick leave*,* then the waiting to hear about whether they’d allow me to stay on sick leave*,* I thought that was really hard” (Woman*,* 50 years).*

Managers felt they had limited possibilities to support the women’s health because of “non-work” aspects, such as the women’s private life and how the healthcare sector works: *“…I want to be able to help them here and now*,* I want to be able to call and say now I want a time for this person*,* so I know I have a deadline*,* so that they themselves*,* when they’re at their worst*,* don’t have to start calling and hunting people down” (First-line manager*,* 58 years)*. Moreover, one manager said that some symptoms were more difficult than others to find a solution for at work, for example, dizziness, brain fatigue, depression, sound sensitivity and emotional vulnerability. Nevertheless, as time went by, the women gradually felt better according to themselves and the managers. Several of them, however, did experience worsening symptoms upon increasing their work hours, which is why the women felt that it was important to have an overcapacity. Managers offered a similar description, i.e., stating that women who increased their work hours were not always ready for it.

Ultimately, the women’s sick leave ended. A few women described their health as good at the time of the interview, but most did not. The remaining symptoms were typically cognitive difficulties, such as feeling that one’s thoughts were slow, that one’s brain could not filter impressions and having difficulties remembering things. Therefore, women feared making mistakes at work or relapsing into sick leave. Because they were no longer on sick leave, women felt that others expected them to be in good health: *“If I’m not 100% healthy… how am I going to produce as if I were 100% healthy? Because that’s what they demanded” (Woman*,* 45 years)*. On the other hand, adjustments at work after the sick leave could lead to feelings of guilt. Most of the women felt compelled to work part-time because of the remaining symptoms, and some were convinced they would have to live with the symptoms for many years to come. The managers were aware of the continued need for support and usually saw part-time work as the solution as well. In some cases, the managers felt the only thing that could improve the women’s health was to change jobs, but they were afraid to bring it up. Instead, they waited for the women to discover this themselves.

#### Trying to find a match between work characteristics and the person

After work resumption, the match between work characteristics and the characteristics of the women was described as important for women’s health. The women talked about what needs they wanted the workplace to satisfy, e.g., having their own office, stability in the coworker group (same people over time), being able to do one work task at a time and having an extra capacity for bad days: “…*thanks to the fact that this job isn’t so stressful I have the time I need to express myself and consider things” (Woman*,* 31 years).* Managers, in turn, described workplace characteristics that facilitated adjustments to women’s needs (without describing *what* needs). They meant that a workplace with a low number of employees per manager, sufficient staff, several units, and several work tasks to choose from – where employees have control over their work situation – was beneficial for health. However, managers described having limited opportunities to adjust the work situation because several issues (e.g., number of staff or geographical location of the workplace) are decided at a higher organizational or political level. Additionally, they felt torn between their personal feelings for the women, the women’s needs, and the organization’s needs. They wanted to adjust the work situation for the women, but not at the expense of colleagues’ health/mood or the women’s ability to perform their usual job: *“…the thing that’s a bit tricky is also that*,* that the adjustments you make*,* they become the new normal” (First-line manager*,* 57 years).* Generally, the managers felt they had more opportunities to make adjustments earlier in the RTW process than later. This was because, earlier in the RTW process, the woman was not considered part of the ordinary work group and, therefore, she could be given any work task without negative consequences for workplace productivity.

Both women and managers described the importance of the “right” work tasks. According to most of the women, relational work tasks were the easiest to perform and beneficial for health: *“…what’s easy is actually having patients… uh*,* because then the focus isn’t on me… I mean*,* then it’s*,* then I can help somebody else*,* with their problem… so that’s really what makes me feel best” (Woman*,* 29 years).* In contrast, staff meetings and administrative work tasks were described as cognitively difficult and therefore detrimental to health. Most managers, in turn, talked about administrative work tasks as something easy, but wanted the women to meet patients anyway so they would get used to their “usual job” as soon as possible. However, one manager said that administrative work tasks were typically the initial work tasks after sick leave: *“…so having patients maybe isn’t the first step when someone returns*,* instead they come back and*,* well*,* do some administrative work” (First-line manager*,* 48 years).*

Unlike the managers, some women highlighted small things in the physical work environment that were stressful, e.g., having to go past the manager’s office every time they wanted to get a cup of coffee, or having a broken lock on the restroom door: “…*there were a lot of these minor stressors*,* like you know*,* the restroom we have*,* before when you locked it you couldn’t see that it was locked… so you always had to… or when you were sitting there somebody could come by and rattle the door handle” (Woman*,* 36 years).*

#### Having to depend on supporters’ personalities and experience

During the RTW process, the women and managers were dependent on others’ support for women’s health improvement. Women mainly received support from their manager, other stakeholders (e.g., healthcare) and people in private life, while the managers mainly received support from the employer support office (e.g., human resources) and from other employees (who took over work tasks). Both the women and managers reported that the degree/kind of support given depended much on the supporters’ personalities and experiences. For example, the women mentioned that they and their manager had a poor understanding of each other’s way of working or thinking: *“She and I work very differently… and that means that she has… very little understanding of me and my way of working” (Woman*,* 40 years).* Another woman said she had seen several physicians and that one had a better dialogue with the Insurance Agency and could therefore put her on sick leave for longer periods. Some of the managers had noticed that the degree/kind of support given by their employer was dependent on work culture or experience: *“…a very inexperienced HR department*,* uh organization… uh*,* it was hard to get any concrete advice” (First-line manager*,* 44 years).* Several women described a high workload at home due to a lack of support from their partners, saying they did not know how to change that. One woman described that her child had special needs at school, but that the assistant and the child did not have good “personal chemistry”, putting an increased practical and emotional burden on the woman.

According to several women, important characteristics among the managers included having knowledge about CMDs, being able to create a pleasant atmosphere at work, being present but not controlling and being willing to have frequent contacts and draw up individualized RTW plans. Like the women, the managers emphasized the importance of their knowledge about CMDs and felt that personal experience of CMDs was a facilitator. Nevertheless, several managers described that they had insufficient knowledge about CMDs and labour law issues regarding RTW. Because of this, they supported the women in the way they thought best or as they had been told to by their employer support, as one relatively new manager said: *“…it feels like the HR world is all about their individual preferences… that’s why I feel like some time in*,* in my life I’m going to get educated and well*,* I’m still kind of in the Bambi phase where I’m just trying*,* uh… to gain experience*,* but at some point I want to understand all this better” (First-line manager*,* 34 years).* Managers also described that their attitude towards CMDs mattered, for example, they felt it was good if they talked a lot about CMDs in the workplace to make the situation less dramatic, or if they viewed sick leave as an opportunity to reveal and attend to the women’s strengths *“…maybe you’re really really good at making implementation plans*,* but you can’t be around people right now… which means that we might have to adjust things” (First-line manager*,* 42 years).*

Women described that some managers were not present, experienced, or attentive enough to notice the women’s state of health, but some of them were. Managers, in turn, said they could determine the women’s health in most cases, but that some women hid how they felt, which made it difficult for the managers to understand their state of health. Therefore, it was important to the managers that the women communicated how they felt: *“…nobody can read minds*,* I can’t know how you feel and you can’t know how I feel… if we don’t actually tell each other” (First-line manager*,* 38 years).* Both women and managers said that changed behaviour (such as suddenly avoiding the coffee room or showing signs of low self-esteem) and lower work performance were common signs of deteriorated health among the women.

#### Relying on women’s use of internal resources

Both the women and managers described women’s use of internal resources as central to improved health in the RTW process. The women mainly described their strategies, while the managers mostly described women’s personality traits. Strategies used by the women included setting boundaries towards others, increasing physical exercise and being responsive to body signals, for example, when the brain feels tired after taking in too many impressions: *“…and then I really have to*,* you know*,* get away for a while*,* put my earplugs in*,* and just like sit in silence and close my eyes… then things can get better” (Woman*,* 41 years).* However, strategies may be easier to use if one’s health is better, resulting in a kind of “Catch-22” situation. Some women decided to change jobs to improve their health. They liked their new workplace better. Most women decided not to tell their new manager about their CMD, because they were afraid their strengths would be overshadowed by the disorder. The remaining symptoms/treatments could therefore be explained by another disorder: *“Then I guess I said that it was partly related to my ADHD*,* that it wasn’t about me being burned out” (Woman*,* 45 years).*

Managers meant that personality traits, essentially the women’s motivation to recover, were important: *“I think it has to do a bit with the person’s attitude… some employees I’ve had well they*,* they’re really like now I’m going to solve this*,* I’m going to take care of it and …. get myself to therapy and really try to change my life because they don’t want to get stuck with poor mental health” (First-line manager*,* 57 years)*. Managers also described that some women want to do more than their health will allow; these women had to be slowed down to prevent deterioration of their situation. Negative personality traits among women, according to managers, were having an unreasonably high sense of responsibility or demands on oneself, identifying themselves with their CMD, introversion, bitterness or being a person who is dissatisfied with everything no matter the external conditions: *“…some employees are really difficult to deal with… regardless of their profession … it’s like*,* nothing is ever good… there’s always something wrong” (First-line manager*,* 43 years).*

### Women’s psychological well-being at work after work resumption

This content area concerned women’s experiences of what contributes to their own psychological well-being at work after work resumption and managers’ experiences of what contributes to women’s psychological well-being at work after work resumption. Psychological well-being at work was defined as feeling happiness, meaning and a sense of being significant at work.

Stable personal conditions.

Having stable personal conditions, i.e., a family situation compatible with work, good health and high self-esteem, were described as important to women’s psychological well-being at work. One woman described feeling that being a single parent and having to work weekends were obstacles to experiencing high levels of psychological well-being: *“…I’m*,* I am at work but thinking about my son*,* he’s at home… and then the limited free time I have*,* I call him or send messages*,* so in one way I’m at work but I’m also at home too*,* because I think that he’s so … sometimes it makes me sad*,* actually” (Woman*,* 48 years).* The managers also mentioned, though only briefly, the importance of having a family life that is compatible with work life.

The importance of good health for psychological well-being at work was discussed by a few women and managers. The managers described it as an absence of symptoms, as one manager said: *“…that you get through a day with having these like symptoms (can result in psychological well-being)” (First-line manager*,* 35 years).* In contrast, the women described health as being able to manage work tasks like others do or like they did themselves before sick leave. Women and managers also described the importance of improved self-esteem, but in different ways. The managers felt they could improve the women’s self-esteem by providing work tasks that corresponded to the women’s capacity. The women, however, highlighted their own attitude towards their achievements at work, for example, thinking that “good enough” is okay or reminding themselves of what they are capable of: *“…I have to remind myself about why I’m here and what I have to offer” (Woman*,* 50 years).*

### Stimulating work tasks

The women and managers said that the women’s jobs provided naturally stimulating work tasks because they could make a difference in other people’s lives. One woman gave an example of a patient encounter that had improved her psychological well-being: ”… *If I anyway made this visit with this mother and her 19-year-old daughter with a mild intellectual impairment and autism a good visit*,* if they left here and felt like oh*,* it was so nice to meet XXX (the interviewee)*,* she listened to us and wants to help us” (Woman*,* 36 years).* The managers felt that thanks to the naturally occurring stimulating work tasks, psychological well-being at work partly came naturally to the women: *“I think that many nurses … uh… sort of get that automatically in their everyday work like*,* you have a patient who’s in pain… and you provide pain relief and that person is happy*,* I think individual nurses get a lot out of that” (First-line manager*,* 34 years)*. According to several of the managers, it was therefore important that the women continued with their usual work tasks and were not given “fictive” administrative work tasks just because they could manage them.

Some of the women gave a more complex picture of the stimulating work tasks, for example, stating that working with patients could be experienced as negative if they had the same patients for a long time (this required more energy than shorter periods of contact) or if the work was perceived as ineffectively organized: *” If you come to me with heel spurs on both feet… then I would treat them at the same time*,* whereas my boss believes you should treat the left foot one day and the right foot another day… and then*,* then my work loses meaning… when I’m not allowed to work in the best possible way… for me and the patient” (Woman*,* 45 years).* A few women and managers also emphasized the importance of professional development and intellectual stimulation at work. The women wanted to learn new things through education, more advanced work tasks, or creative discussions and have a feeling of continuously moving forward. However, one manager said that allowing this was risky: *”… it’s really a delicate balance because it’s easy for the situation to become… uh… to become too stressful” (First-line manager*,* 48 years)*.

### Strengthening relationships at work

The women and managers stressed that strengthening relationships in the co-worker group is essential to promoting women’s psychological well-being at work. According to the women and managers, the women needed to feel included in a positive social context at work, one dominated by benevolent relationships. However, while the managers talked more about making the women feel included at work in relation to being an essential part of the working team and getting the same information as everyone else, the women instead emphasized the importance of having fun with colleagues during the workday, for example, taking coffee breaks or eating lunch together without having to rush, or just joking with each other. One woman described that one of her colleagues had also experienced CMD and that they joked about it: *” Sometimes we go into each other and say*,* now I have no anxiety*,* isn’t there something we can worry about today? Yeah there is*,* sure there is… so actually we’re having quite a good time” (Woman*,* 53 years).*

Furthermore, according to women and managers, the women had to feel appreciated for their work performance, despite shortcomings or “bad days”, as one manager used to say: *“…it’s completely okay*,* we’ll start fresh tomorrow” (First-line manager*,* 51 years)*. Unlike the managers, the women said it was important that managers showed an interest in employees’ psychological well-being and not only in the physical work environment or production rates. They emphasized the importance of being valued as individuals, not only as workers, as one woman said: *“…I want to live in a community where people really count… I mean we should feel good regardless*,* I’m not just what I produce” (Woman*,* 40 years).*

## Discussion

The present results showed that, according to the women and the first-line managers, women’s health and psychological well-being during the RTW process depend on the individual characteristics of women themselves, their private life, work and other RTW stakeholders. The process of health improvement went up and down, and the women’s state of health was usually still fragile after the end of sick leave. The women and the managers had different perceptions of some things, such as what work tasks were easiest for the women to perform immediately after work resumption. Moreover, the women described minor stressors at work that the managers did not talk about. Regarding psychological well-being at work after work resumption, both the women and managers described that it partly comes naturally in caring professions given the inherent meaningfulness of helping others. However, the women felt that, for caring situations to be meaningful, certain working conditions were a prerequisite. Both the women and managers described that having work that was compatible with private life, being in good health and having strengthening relationships at work were necessary for the women to experience high levels of psychological well-being at work. The women emphasized having fun with colleagues and being seen as a person (not only “a worker”), while the managers talked more about women’s need for inclusion and tolerance at work.

Overall, the individual characteristics of the women themselves, RTW stakeholders, private life and the workplace seemed to be the core aspects regarding the improvement of women’s health as well as their psychological well-being at work. In previous research, a multidisciplinary approach involving work and healthcare professionals has been commonly used in RTW interventions [11, 50, 51], but the present results indicate that an even more holistic approach should be considered, i.e., including women’s private life (e.g., family). It is known that significant others influence RTW beliefs among individuals with chronic disorders [52]. Also, Nybergh et al. [53] study indicated a need for home-related aspects in future RTW interventions among women with CMDs. However, women’s health and well-being is not just an individual issue. Organisational factors controlled by management and policy should not be overlooked as they underpin workplace conditions the foundation for the conditions in the workplace.

The women’s health did improve during the RTW process. We cannot discern, however, whether that was because of their own strategies, received support, time passing, or all of these factors. Increased work hours were described as a risk for deteriorated health because of the increased demands. Previous research has shown similar results, i.e., that increasing working hours can be detrimental to health [30]. Skärsäter and Willman [54] claimed that regaining health after depression includes so-called “critical points”, i.e., events that entail insecurity, increased demands and therefore an increased risk of relapsing into worsened health. Increasing work hours could also be seen as one of those critical points. When increasing working hours, RTW stakeholders should pay extra attention to the employee. Managers in the present study mentioned the importance of women communicating verbally about how they felt, which might be even more important during these sensitive periods as regards decreasing the risk of misinterpretations. As RTW is a complex issue, women and first-line managers should be supported by other RTW stakeholders (e.g., human resources or occupational health services) to protect women’s health when workloads increase.

Both the women and managers felt it was important for managers to have sufficient knowledge about CMDs. Nevertheless, managers without personal experience of CMDs (having been ill themselves) experienced a lack of knowledge in this area. A lack of knowledge regarding CMDs has also been described in previous research [14, 46]. Moreover, several managers in the present study described that they lacked knowledge about rights and obligations in the RTW process in general even though the vast majority had attended leadership training. This may indicate that more education on CMDs and the RTW process should be offered to managers regarding both “hard facts” (e.g., obligations) and more “soft facts” (e.g., “Can I say to the employee that I don’t think this job is right for her?”). Alternatively, or in addition, support from professionals with more knowledge of RTW and CMDs should be accessible to managers.

All women and managers in this study worked within female dominated professions with known emotional stressors and organizational shortcomings [11]. Changing jobs was sometimes necessary for the women to improve their health, according to both the women and managers. In previous research, employees going through the RTW process after CMDs have described that liking their jobs facilitated working (and vice versa: not liking their job was a barrier) [22]. Almost all women in the present study who had changed jobs had chosen a job similar to the one they had before. Thus, despite having experienced a CMD partly due to work-related issues, they wanted to stay in the same sector. The women described that relational work tasks were the easiest and most meaningful to perform after work resumption. This is surprising because it is not in line with previous research. Studies have shown that relational work tasks are problematic for individuals with symptoms of CMDs [55, 56]. The managers in the current study did however not talk about relational work tasks in a positive manner regarding health promotion. Rather, they talked about such tasks as something the women had to get used to. This means there may be a discrepancy between the women’s and managers’ perception of the importance of relational work tasks for health, and this discrepancy may form a breeding ground for misunderstandings when planning the women’s RTW.

Most women were still experiencing deteriorated health even though one month or more had passed since the end of sick leave. Some women believed that their symptoms would remain for a very long time. Previous research has shown that nearly half of those who have had a stress-related disorder experienced symptoms such as fatigue and cognitive difficulties seven years later, and almost three-quarters reported decreased tolerance for stress [57]. Given the protracted nature of these symptoms, it may be important for managers and women to focus on improving psychological well-being at work. However, the organizational climate and support functions need to be aligned. According to previous research among other groups, having a high level of psychological well-being may help employees enjoy better health [34, 58]. Moreover, based on the present results, it seems to be important for women to tell managers about minor stressors in the work environment. Such minor stressors were only mentioned by the women, indicating that this may be an aspect that is overlooked by or unknown to managers.

The women and managers described those strengthening relationships at work as important for women’s psychological well-being. However, there were differences between the women’s and managers’ descriptions. Generally speaking, the women wanted to have fun with colleagues and be appreciated as unique individuals, while the managers focused on inclusion and tolerance for limitations. The manager’s descriptions were in line with the focus of previous research on psychological well-being at work [59, 60], i.e., that psychological well-being can be increased by eliminating stressors rather than by adding positive things. The women’s descriptions, however, were in line with Corbières et al.’s study [61], which showed that employees felt that recognizing one’s own worth is important in promoting sustainable RTW after CMDs. Based on this, managers could contribute to a good work environment by creating opportunities for informal social interactions at work and together with the employees striving for a working climate where everyone is valued for their uniqueness and not only for being workers. They need, however, organizational prerequisites for this. Future research should focus on obtaining a more comprehensive picture of psychological well-being at work. For example, do employees experience high levels of psychological well-being at work after work resumption? What role does the first-line manager play in promoting psychological well-being at work? What barriers to and facilitators of psychological well-being at work exist?

### Methodological considerations

The study has some limitations. First, most questions posed to the women were retrospective, which might have caused some memory bias [62]. Moreover, all women had been on full-time sick leave sometime during the RTW process. The transferability of the results to women who have only been on part-time sick leave, and therefore experienced another kind of RTW process, is therefore limited. The transferability to men and employees in other professions than healthcare, social care and schools is also limited because the study only includes women in welfare sectors. However, sick leave for CMDs is common among these professions internationally and in Sweden [10, 63]. Regarding trustworthiness [64], it is considered to be a strength that pilot interviews were conducted and that women were asked to suggest questions to the managers’ interview guide. This may enhance the relevance of the questions. It can however be considered to be a weakness that the managers were not asked to suggest items to the women, which was a consequence of the women being interviewed first so the managers later could be matched to their professions. It is however a strength that the research group took part in the interviews and was involved in the data analysis. Also, women and managers had varying characteristics (e.g. age, diagnosis, experience) which increased the likelihood of covering the breadth of the phenomenon under study. A weakness is though that the managers were recruited from a smaller and more rural area than the women, meaning that they did not correspond to each other fully regarding geographical context. The convenience sampling of the women, instead of matching them with their managers, was chosen to minimize the risk of any participant feeling uncomfortable. Furthermore, the periods of sick leave that women and managers referred to in the interviews largely took place during the Covid-19 pandemic, which compromises the transferability of the results to other times. A large number of women during/after long-term sick leave in Sweden reported that the pandemic affected their general well-being and RTW [29].

## Conclusion

Women and first-line managers had mostly similar descriptions. The promotion of women’s health and psychological well-being during the RTW process in female-dominated occupations requires individual assessments/actions as the characteristics of the women involved in relation to their work and their overall life situation are unique in this case. Unfortunately, there is a risk that the administrative RTW process will interfere with women’s own process of health improvement. Although the responsibility for women’s health in female-dominated occupations extends beyond the employees and managers, it is valuable for them to be aware of that relational work tasks can also maintain or improve women’s health and that minor stressors in the work environment can reduce it. In addition, managers would probably benefit from more knowledge about CMDs and the rights and obligations involved in the RTW process. With regard to women’s psychological well-being at work after returning to work, more research is needed to investigate how it can be maintained or increased.

## Data Availability

The datasets used and/or analysed during the current study are available from the corresponding author upon reasonable request.
